# Immune-Mediated Desquamative Gingivitis and Optical Coherence Tomography Diagnostic Patterns: Clinical Implication from a Systematic Review

**DOI:** 10.3390/diagnostics11081453

**Published:** 2021-08-11

**Authors:** Vera Panzarella, Alessia Bartolone, Vito Rodolico, Giorgia Capocasale, Laura Maniscalco, Domenica Matranga, Olga Di Fede, Giuseppina Campisi

**Affiliations:** 1 Department of Surgical, Oncological and Oral Sciences, University of Palermo, 90127 Palermo, Italy; alessia.bartolone@community.unipa.it (A.B.); olga.difede@unipa.it (O.D.F.); giuseppina.campisi@unipa.it (G.C.); 2 Department ProMISE, University of Palermo, 90127 Palermo, Italy; vito.rodolico@unipa.it; 3 Section of Dentistry and Maxillofacial Surgery, Department of Surgical Sciences, Paediatrics and Gynecology, University of Verona, 37134 Verona, Italy; capocasalegiorgia@gmail.com; 4 Department of Biomedicine, Neuroscience and Advanced Diagnostics, University of Palermo, 90127 Palermo, Italy; maniscalco.laura92@gmail.com; 5 Department of Health Promotion, Mother and Child Care, Internal Medicine and Medical Specialties, University of Palermo, 90127 Palermo, Italy; domenica.matranga@unipa.it

**Keywords:** desquamative gingivitis, optical coherence tomography, oral pemphigus vulgaris, oral mucous membrane pemphigoid

## Abstract

Desquamative Gingivitis (DG) comprises heterogeneous clinical manifestations of numerous immune-mediated muco-cutaneous diseases. Optical Coherence Tomography (OCT) has been proposed as a valuable diagnostic support even if, to date, there are no standardized OCT-diagnostic patterns applicable to DGs. A systematic review was performed to detect existing data on in vivo OCT diagnostic patterns of the most common immune-mediated DGs (i.e., pemphigus vulgaris, mucous membrane pemphigoid and oral lichen planus). It has been found that OCT exhibits specific patterns that address the diagnosis of DG by pemphigus vulgaris (i.e., intraepithelial unilocular blister, reduced epithelial thickness, presence of acantholytic cells in the blister) and by mucous membrane pemphigoid (i.e., subepithelial multilocular blister, presence of inflammatory infiltrate), but not by oral lichen planus. These patterns could offer an attractive diagnostic OCT framework to support the clinical preliminary assessment and monitoring of these complex pathological conditions.

## 1. Introduction

Desquamative gingivitis (DG) denotes a clinical sign of a very large spectrum of diseases with different pathogenesis [1]. Among these, Pemphigus Vulgaris (PV), Mucous Membrane Pemphigoid (MMP) and Oral Lichen Planus (OLP) represent about 80% of cases of DG [1,2]. These immune-mediated diseases could be characterized by muco-cutaneous involvement and chronic course. However, almost one-third of the patients presented primarily with only gingival involvement in the form of DG which remains underdiagnosed for a longer time than in cases of multisystem involvement [3]. The reason for this evidence is not known, but it could be attributed to the non-specific features of immune-mediated DG compared to many conditions that present as gingival inflammation, especially those that are plaque-related.

As a consequence, efforts to improve their early detection are mandatory [4]. To date, the diagnostic algorithm for immune-mediated DG includes the combination of clinical/anamnestic data (e.g., clinical onset, extra-oral involvement, Nikolsky’s sign), immuno-serological tests (i.e., indirect immunofluorescence, enzyme-linked immunosorbent assay) and histological examinations [4,5]; particularly, direct immunofluorescence (DIF) is considered the gold standard approach [2,3,6,7]. However, DIF is an expensive technique, available only in advanced research laboratories [8]; also, the practice of biopsy in gingiva affected by DG is difficult because the tissues are fragile and thin, and so are difficult to manipulate [5].

Among new optical imaging technologies, Optical Coherence Tomography (OCT) is playing an increasingly important diagnostic role in several areas of medicine. This tool provides high-resolution, micron-scale tomographic images of the microstructure of different tissues [9]; therefore, it could be an excellent non-invasive support in oral medicine, especially for the diagnosis and management of patients with oral chronic disease [10,11,12], such as DG. However, to date, the OCT interpretation of oral tissue remains highly operator sensitive, and it lacks a standardization of OCT parameters to perform diagnostic in vivo evaluation of DGs. 

This commentary describes a systematic review of the most discriminatory OCT patterns with respect to the three most frequent DG immune-mediated diseases, such as PV, MMP and OLP. The principal aim is providing standardized OCT diagnostic models to support their non-invasive clinical diagnosis and chronic monitoring. 

## 2. Materials and Methods

The Preferred Reporting Items for Systematic Reviews and Meta-Analyses (PRISMA) standard was used for this systematic review. A selection of the studies concerning the use of OCT in patients with DGs (by MMP, PV and OLP) was performed. A comprehensive search of electronic databases (PubMed, PubMed Central/PMC) was conducted, using the following search terms: optical coherence tomography/OCT, desquamative gingivitis/DG, oral pemphigus, oral mucous membrane pemphigoid, oral lichen planus. 

The inclusion criteria chosen were in vivo studies investigating oral PV, MMP, OLP by OCT and English studies published from 2005 to 2021. Review articles, commentaries, conference abstracts, opinion articles and letters to the editor were excluded. The studies were initially selected by title and abstract. Duplicate papers were removed, and selected articles were scrutinized to assess for eligibility. Research was completed in April 2021. 

Each paper included in this systematic review was structured according to the patient Population, Intervention, Comparison, Outcomes and Study design (PICOS) method. The risk of bias was assessed according to the Risk of Bias In Non-randomized Studies of Intervention (ROBINS-I) tool [13]. The overall risk of bias for each study is defined as low or moderate if all domains are at low or moderate risk, respectively. Otherwise, if at least one domain is serious or critical, the overall risk of bias of the study will be judged serious or critical, respectively. Quantitative variables were mean and standard deviation; qualitative variables were count and percentage.

## 3. Results

**Figure 1:**
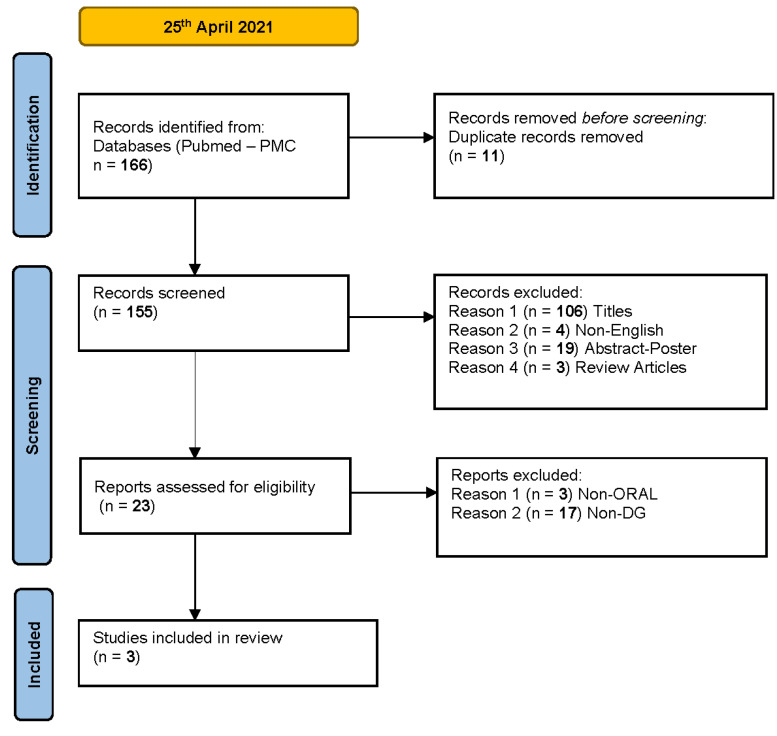
The flowchart summarizes the steps in the selection process for systematic review.

The initial literature search identified a total of 166 citations (Figure 1). 

Duplicate articles were removed (11), leaving 155 for screening. Articles were rejected if the title was not appropriate, they were not in English, or if they were abstract-poster or review articles (132). The papers assessed for eligibility were 23, but 20 were excluded because they were not oral and/or not DG. Only three articles met our inclusion criteria: two case-reports and one case-series of three patients. 

The PICOS information about the three selected papers and their contents is given in Table 1. 

The overall risk of the selected studies was judged as severe because of at least one severe risk (bias in participant selection) (Table 2).

Five subjects from the three selected papers had average age of 62.6 ± 3.65 years (range 57–67); 60% were diagnosed with MMP (average age 62.67 ± 5.13 years) and the remaining 40% with PV (average age 62.5 ± 0.70 years). All cases (100%) of PV patients had intraepithelial blisters, with a unilocular morphology reported in one case of two (50%) investigating this parameter. Of the MMP patients, 100% had a subepithelial blister, with a multilocular pattern reported in one of three cases (33.3%) investigating blister morphology. The epithelial thickness was considered in 50% of PV cases and in 66.67% of MMP cases, respectively reporting a reduced vs. a normal pattern. A normal/homogeneous status of basal membrane and presence of inflammatory infiltrate were detected, respectively, in one investigated case of two PV patients and in two investigated over three MMP cases (50% and 66.67%, respectively). The presence of acantholytic cells in the blister was detected in one of the two investigated PV cases (50%).

## 4. Discussion

The “diagnostic gold standard” algorithm for desquamative gingivitis includes histopathology, direct immunofluorescence (DIF) and immuno-serological investigations [2,4]. However, the diagnostic delay related to immune-mediated DGs is currently long, with a significant increase in patients who had DGs as initial/unique manifestation [3]. 

Consequently, inappropriate treatment and multiorgan involvement frequently occur, increasing prolonged patient suffering and impaired quality of life [3,14]. In this context, OCT could be considered as a promising tool to support the in vivo preliminary evaluation of the more frequent immune-mediated diseases associated with DG, such as pemphigus, mucous membrane pemphigoid and lichen planus.

**Figure 2:**
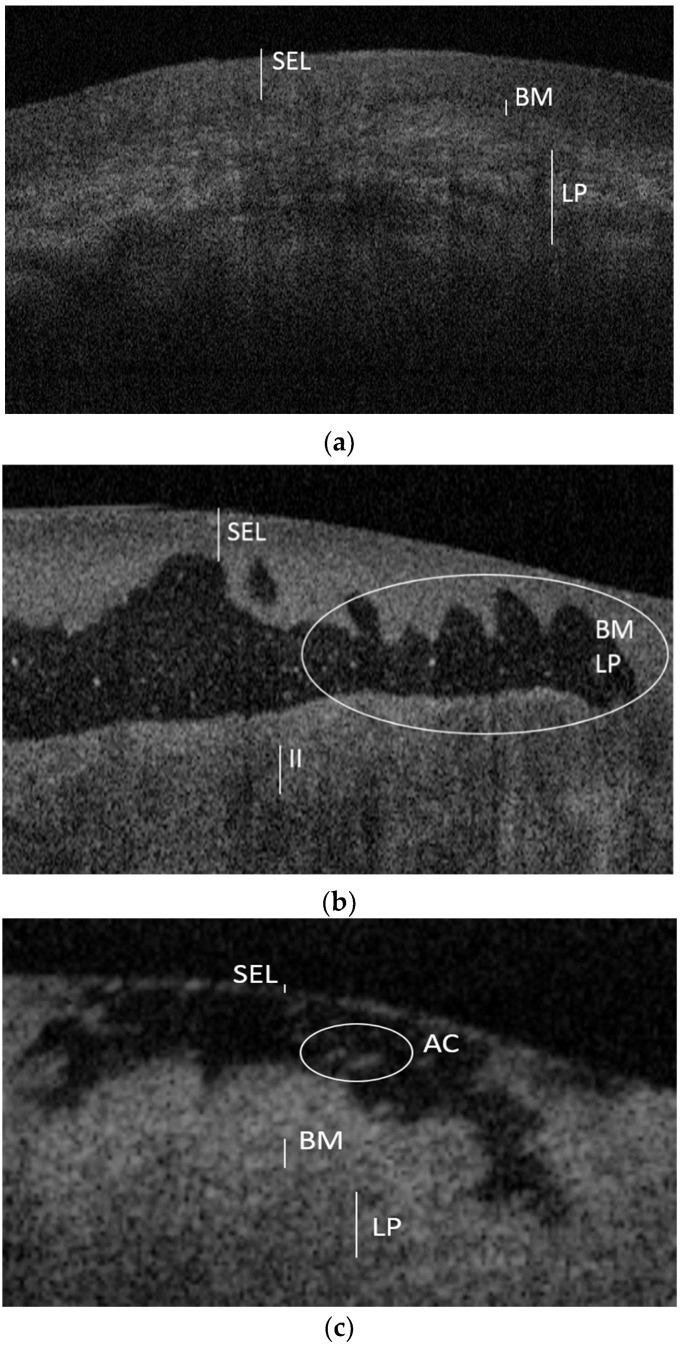
OCT images of normal gingival mucosa (**a**), gingival mucosa with MMP (**b**) and gingival mucosa with PV (**c**). Compared to the healthy mucosa (**a**), in the DG by MMP (**b**) there is the presence of a multilocular subepithelial blister, together with a dense inflammatory infiltrate (II) underlying an altered profile of both the basement membrane (BM) and the lamina propria (LP). In the DG by PV (**c**) there is a unilocular intraepithelial blister which reduces the thickness of the stratified epithelial layer (SEL); it is also possible to observe acantholytic cells (AC) inside the blister.

The main finding of this study was to survey specific standardized diagnostic patterns for MMP and PV immune mediated DGs, summarized below (and in Figure 2).

**OCT pattern for MMP:**

- Presence of multilocular subepithelial blister
- Normal stratified epithelial layer and epithelial thickness
- Altered/indistinguishable basal membrane and lamina propria
- Presence of inflammatory infiltrate

**OCT pattern for PV:**

- Presence of unilocular intraepithelial blister
- Reduced stratified epithelial layer and epithelial thickness
- Normal basal membrane and lamina propria
- Presence of acantholytic cells in the blister

The above findings are limited by the small sample size available, probably reflecting the rarity of these diseases. No specific OCT patterns have been found for DG related to OLP.

## 5. Conclusions

In conclusion, this systematic review found specific OCT diagnostic patterns for preliminary diagnosis of DG related to pemphigus and mucous membrane pemphigoid diseases. In the future, they could offer an attractive diagnostic OCT framework to support clinical preliminary assessment and monitoring of these complex pathological conditions, as well as the creation of specific OCT software, able to perform a standardized digital diagnosis. 

## Figures and Tables

**Table 1:** PICOS details of the three selected studies.

First Author (Year)	Population (Disease)	Intervention (Type, Model, Brand, Device)	Outcomes	Study Design	OCT Diagnostic Parameters	Reference Diagnosis
Di Stasio (2015)	1 case (PV)	NS	Evaluation of feasibility to image epithelial architecture of oral PV in vivo	Case report	− Localization of blister (intraepithelial vs. subepithelial) − Status of the basal membrane (MB) (normal/homogeneous vs. alterated/indistinguishable)	None
Capocasale (2018)	1 case (MMP)	SS -OCT VivoSight® Michelson Diagnosis Ltd, version 2.0, Orpington, Kent, UK	Evaluation of tissue microstructure in a patient with oral MMP	Case report	− Localization of blister (intraepithelial vs. subepithelial	Histopathology
Di Stasio (2020)	3 cases (1 PV, 2 MMP)	SS- OCT (IVS- 300 by Santec)	Examination of epithelial and subepithelial layer, and distinction between intra-and sub-epithelial detachment in oral PV and MMP lesions	Case series	− Localization of blister (intraepithelial vs. subepithelial) − Morphology of blister (unilocular or multilocular) − Epithelial thickness (normal vs. reduced) − Acantholytic cells into blister (present vs. absent). Only for PV case − Inflammatory infiltrate (present vs. absent). Only for MMP cases	Histopathology and DIF

NS, Not Specified; SS, Swept-Source; OCT, Optical Coherence Tomography; MMP, Mucous Membrane Pemphigoid; PV, Pemphigus Vulgaris; DG, Desquamative Gingivitis; DIF, Direct Immunofluorescence.

**Table 2:** ROBINS-I assessment for the three selected studies.

Observational Studies	Bias Due to Confounding	Bias in Participant Selection	Bias in Classification of Interventions	Bias Due to Deviation from Intended Interventions	Bias Due to Missing Data	Bias in Measurement of Outcomes	Bias in Selection of the Reported Result	Overall Bias
Di Stasio (2015)	UR	SR	LR	NI	LR	MR	LR	SR
Capocasale (2018)	NI	SR	LR	NI	LR	MR	LR	SR
Di Stasio (2020)	NI	SR	LR	NI	LR	MR	LR	SR

Low risk (LR), moderate risk (MR), serious risk (SR), and critical risk (CR) or not interpretable (NI).

## Data Availability

Data available on request.
